# Antianemic activity of quinoa (*Chenopodium quinoa* Willd) Collana Negra variety and kanihua (*Chenopodium pallidicaule* Aellen) Ramis variety seed flour in anemic rats

**DOI:** 10.1007/s42452-022-05202-w

**Published:** 2022-10-23

**Authors:** Gladys Moscoso-Mujica, Ángel Mujica, Juana Chávez, Carmen Peña, Noelia Begazo, Jumira Estrella, Zaira Estrada, Liliana Tello, Yeltsin Ramos, David Rivera, Carla Inocente, Fabricio Huarca

**Affiliations:** 1 Research group of Experimental Pharmacology - Faculty of Pharmacy and Biochemistry, Norbert Wiener Private University, Lima, Peru; 2 Research group of Toxicological Biochemistry - Department of Biochemistry - Faculty of Pharmacy and Biochemistry, National University of San Marcos, Lima 1, Peru; 3 Department of Biochemistry - Faculty of Pharmacy and Biochemistry, National University of San Marcos, Lima 1, Peru; 4 Postgraduate School, National University of the Altiplano, Puno, Peru; 5 Postgraduate in Enviromental Sciences, Catholic University of Santa Maria, Arequipa, Peru

**Keywords:** *Chenopodium quinoa* Willd, *Chenopodium pallidicaule* Aellen, Anemia, Antianemic activity, Hematocrit

## Abstract

The Andean grains from the Peruvian Altiplano, quinoa (*Chenopodium quinoa* Willd) and kanihua (*Chenopodium pallidicaule* Aellen) have high protein content and an optimal balance of essential amino acids and minerals such as iron (19.8 mg/100 g y 17.6 mg/100 g, respectively). The objective of this research was to evaluate the antianemic activity of extruded flour from quinoa seeds variety Negra Collana and kanihua variety Ramis in anemic *Holtzman* strain rats. The results of the proximal analysis showed high protein content in quinoa at 22% and kanihua at 16.2%, and the acute toxicity test showed harmlessness up to the dose of 15000 mg/Kg in both flours confirmed with the anatomopathological observation of organs such as liver, stomach, lung, kidneys, and brain. In the evaluation of the antianemic activity, a basal average of 29.3 ± 0.2% of hematocrit was observed in the group of anemic rats treated with quinoa flour, and in twelve weeks, it increased to 53.8 ± 0.3% of hematocrit (*p* ≤ 0.05). A group of anemic rats treated with kanihua flour had a basal average of 29.5 ± 0.3%, and in twelve weeks, it increased to 51.7 ± 0.3% (*p* ≤ 0.05). A group of rats without anemia treated with quinoa and kanihua flour showed a basal average of 50.2 ± 0.2% and 49.3 ± 0.3%; in twelve weeks, it increased to 55.2 ± 0.2% and 54.8 ± 0.1%, respectively. It was concluded that oral administration of 360 mg/Kg every 24 h of quinoa flour and kanihua flour increased hematocrit levels by 24.5 ± 0.5% and 22.2 ± 0.3%; weight 65.8 ± 0.3 g and 59.2 ± 0.1 g; height 6.8 ± 0.1 cm and 5.7 ± 0.5 cm, respectively (*p* ≤ 0.05). In rats without anemia increased hematocrit levels by 5.3 ± 0.0% and 5.5 ± 0.0%; weight 37.7 ± 0.1 g and 21.7 ± 0.05 g; height 4 ± 0.0 cm and 3.9 ± 0.0 cm, respectively (*p* ≤ 0.05).

## Highlights

- Extruded quinoa flour of the Negra Collana variety and kanihua variety of Ramis are novel nutraceutical ingredients in food design and antianemic of food origin.
- Extruded flour of quinoa variety Negra Collana and kanihua variety Ramis at a dose of 360 mg/kg every 24 h orally for twelve weeks showed a significant increase in hematocrit levels of 24.5 ± 0.5% and 22.2 ± 0.3%, respectively (*p* ≤ 0.05) in anemic rats.
- Extruded quinoa flour of the Negra Collana variety and kanihua variety Ramis showed improvement in the signs and symptoms of anemia such as lethargy, hair loss, apathy, and lack of appetite, weight, height, and BMI in anemic rats. Increased weight, height, BMI, and motor activity in rats without anemia.

## Introduction

Anemia is a disease caused by hemoglobin deficiency that varies according to age and sex; the diagnosis is made through a blood test; the leading cause of anemia is produced by iron deficiency which causes more than 50% of cases of anemia in women and children [1]. Although the primary cause is iron deficiency, it is seldom present in isolation; more frequently, it coexists with many other causes, such as malaria, parasitic infection, nutritional deficiencies, and hemoglobinopathies [2]. There are different types of anemia, such as those caused by vitamin B12 and folic acid deficiency, chronic disease of prolonged conditions that involve inflammation, and others by hemolysis of red blood cells. Also, idiopathic aplastic anemia in which the bone marrow produces insufficient blood cells, pernicious when vitamin B12 is not adequately absorbed; other anemia are transmitted from parents to children, such as sickle cell anemia caused by hemoglobin S and thalassemia due to inadequate hemoglobin production [1–3]. The World Health Organization (WHO) indicates that anemic children have significant emotional, cognitive, and motor deficiencies. Therefore, anemia will severely affect their cognitive performance and growth during adolescence and youth. In pregnant adolescents, anemia causes an increase in maternal morbidity and mortality, with a high incidence of low birth weight and prematurity of the newborn [1, 2]. The patient with mild anemia without other alteration is asymptomatic but may present palpitations, a slight increase in dyspnea, and sweating that daily accompany exercise; when the anemia becomes severe, these symptoms are accentuated as dyspnea to a greater degree, palpitations and throbbing headache, and the ability to work, study or perform other physical activities is reduced [2, 3]. Also, sodium retention has been described as causing a lack of appetite, edema in lower body areas, and secondary indigestion due to the deficient supply of oxygen to the intestine [2, 4]. Accompanied by dizziness, generalized weakness, and sometimes syncope related to compromised vasomotor regulation, central nervous system hypoxia symptoms were causing drowsiness, inability to concentrate, and even disorientation [3]. On physical examination, the anemic patient is pale due to the relationship between the color of the skin and mucous membranes due to the low concentration of hemoglobin [3, 4].

The Andean zone of Peru is one of the eight centers of domestication of cultivated plants in the world, where various grains recognized for their nutritional quality is grown [5]. In the Altiplano of Peru and Bolivia, Andean grains such as quinoa (*Chenopodium quinoa* Willd) and kanihua (*Chenopodium pallidicaule* Aellen), belonging to the *Amaranthaceae* family, are grown [5, 6]. They are the only areas where the diversity and variability of these grains are found in situ, and they are called Aynokas [6]. They present wide adaptation to adverse agroecological conditions such as in highland ecological zones up to 3500–4200 m, tolerance, and resistance to drought, cold, salinity, excess moisture, radiation, among other characteristics [7]. The Food and Agriculture Organization of the United Nations (FAO) [8] points to these Andean grains as biologically complete proteins because they contain the complete essential amino acids in proportions equal to or even higher than what is established for each amino acid in a protein pattern protein [9]. Thus, they are considered complete grain proteins with a wide range of nutrients and phytochemicals that have gained worldwide attention [10–12]. Have a variety of secondary metabolites with a broad spectrum of bioactivities [13]. Quinoa has been cultivated since pre-Inca times, known as quiuna in the Quechua language, which means tight panicle, and in Aymara language, Jiura, or Jhupa, which means raises the dead; it is a functional food with a high nutritional quality that is used in totality the plant [5]. It is entirely due to the ideal balance of its essential amino acids, such as the content of threonine with 3.4 mg/16 g N, lysine 5.6 mg, methionine 3.1 mg, tryptophan 1.1 mg, histidine 2.7 mg, isoleucine 3.4 mg, leucine 6.1 mg, phenylalanine 3.7 mg, valine 4.2 mg; content of balanced fatty acids such as omega 3, 6 and 9; minerals such as iron 19.8 mg/100 g, calcium 94 mg, among other minerals; and vitamins such as riboflavin (B2) 3.9 ppm/dry matter, ascorbic acid 49 ppm, alpha-tocopherol 52.3 ppm, carotenes 5.3 ppm, among other vitamins; dietary fiber content 10.3 g/100 g [7, 8, 12]. On the other hand, the Andean grain kanihua is less widespread and studied than quinoa. It also has high protein quality with an optimal content of essential amino acids such as threonine with 4.7 mg/16 g N, lysine 5.7 mg, methionine 3.0 mg, tryptophan 0.9 mg, histidine 2.7 mg, isoleucine 6.4 mg, leucine 6.1 mg, phenylalanine 3.5 mg, valine 4.5 mg; content of essential fatty acids and omega 3, 6 and 9 in a balanced way, minerals such as iron 17.6 mg/100 g, calcium 171 mg, phosphorus 387 mg; and vitamins such as riboflavin (B2) 0.70 mg/100 g, ascorbic acid 2.2 mg, alpha-tocopherol 22.0 mg, carotenes 2.0 mg; dietary fiber content 11 g/100 g; and it is characterized by having saponins without a bitter taste, unlike quinoa [8, 14, 15]. Both dicotyledonous grains have the minimum amount of the proline protein fraction, and unlike cereals, therefore, it is an ideal food for celiac patients (gluten-free) [16]. In addition, they present bioactive peptides with various pharmacological activities such as antimicrobial, antioxidant, and other activities [17, 18], are considered as functional foods and nutraceuticals. The objective of this research was to evaluate the antianemic activity of extruded flour from seeds of quinoa (*Chenopodium quinoa* Willd) variety Negra Collana and extruded flour from seeds of kanihua (*Chenopodium pallidicaule* Aellen) variety Ramis in anemic *Holtzman* strain rats. That will provide a natural alternative with accessibility in production, elaboration, preparation, and cost in different populations with anemia.

## Materials and methods

### Obtaining the raw material

Quinoa seeds of the Negra Collana variety and kanihua seeds of the Ramis variety were collected in Puno—Peru at 3800 m altitude from the National Institute of Agrarian Innovation (INIA)—ILPA. They were cleaned, ground, extruded to partial removal saponins, and sieved until obtaining 0.250 mm particles, placed in amber bottles, and stored at 4 °C.

### Proximal composition

The analysis of the proximal composition of the extruded quinoa flour and the extruded kanihua flour was carried out at the Analytical Control Center-UNMSM, Lima, Peru; according to the official AOAC procedures [19]. Protein (method 920.53) was calculated according to the Kjeldahl system. Moisture (945.15) is based on the difference in weight when dried in an oven at 104 °C for a period of 2 h. The ash (923.03) was calculated by the weight of the sample after being heated to 550 °C for a period of 2 h. The lipid (920.39B) was extracted with petroleum ether over a period of 4 h in the Soxhlet system. The fiber (962.09) was analyzed by digestion with sodium hydroxide and sulfuric acid solutions and calcination. Carbohydrates were determined by calculation.

### Experimental animals

The experimental animals were male and female *Holtzmann*- source strain albino rats, healthy with an average weight of 250 g, 3–4 months old, from the National Institute of Health of Lima-Peru. They were kept in the animal bioterium of the Faculty of Pharmacy and Biochemistry of the Norbert Wiener Private University, Lima, Peru, at a temperature of 22–24 °C, with photoperiods of 12 h of light/darkness and free access to balanced food (pellets rats) and water. The research was carried out following the provisions of the guide for the care and use of laboratory animals [20].

### Determination of the acute toxicity of the quinoa flour of the Negra Collana variety and the kanihua flour of the Ramis variety

With quinoa flour, 10 groups were formed (n = 3), with kanihua flour 10 groups (n = 3), 9 groups of each flour were administered orally through orogastric cannula different doses of flours of 500, 1000, 3000, 5000, 7000, 9000, 11000, 13000, and 15000 mg/Kg of the weight of the experimental animal, dissolved in physiological solution (NaCl 0.9%) (Gibbco, USA). The control groups were administered only physiological solution (NaCl 0.9%). After 12 h of the administration of the doses, all experimental animals received their balanced food and water in the usual way; the observations of the autonomic and neurological behavior of the rats were made every 2 h for one day and later every 8 h until the seventh day [16].

### Pathological analysis

It was performed by the department of clinical pathology of the Archbishop Loayza National Hospital, Lima, Peru. After concluding the acute toxicity test, euthanasia was performed with an overdose of sodium pentobarbital 50 mg/Kg intraperitoneally; death was confirmed by observing the loss of the pedal reflex, pupillary response to light, and cardiac palpitations. Then necropsies and macroscopic descriptions of the different organs of the experimental animals were performed, such as liver, stomach, lung, kidneys, and brain, and their weight was recorded. The organs were collected in individual containers to be fixed in 10% formalin in 0.1 M phosphate buffer (pH 7); then included in paraffin, the microtomy was performed with a thickness of 4–5 mm, successive compound staining with hematoxylin–eosin and mounting for the performance of histopathological studies [21].

### Induction of experimental anemia

Anemia was induced according to the bleed-out method reported by Delwatta et al*.* [22] with modifications. One mL of blood was drawn from the lateral saphenous vein of the rat three times a week for eight weeks. Anemic rats were considered when they presented hematocrit levels < 33% and hemoglobin < 11 g/dL [23].

### Hematocrit and hemoglobin determination

Hematocrit (Hct) was determined according to the microhematocrit method or percentage globular volume reported by Márquez and Chacón [24] with modifications. The blood was collected in 75 µl heparinized capillary tubes, two-thirds of the capillary was filled at the unmarked end, the opposite distal side was sealed with plasticine at 4 mm, and it was centrifuged in a microcentrifuge (Hettich, Germany) at 11000 rpm for 10 min. Then, a microhematocrit reader (Table cryptocapsTM, Oxford, McCormick Scientific, USA) was used to perform the readings of the heights of the erythrocyte volume and the total volume of the sample. Due to its standardization and reproducibility, this manual method is considered the best parameter to determine anemia. Microhematocrit was calculated as follows:

$${\text{Hematocrit}}\,(\% ) = \left[ {{\text{Height}}\,{\text{ of}}\,{\text{ erythrocyte}}\,{\text{ volume}}\,{\text{(mm)/Height}}\,{\text{ of}}\,{\text{ total}}\,{\text{ volume}}\,{\text{(mm)}}} \right] \, \times 100$$

Hemoglobin (Hb) was calculated according to Rodak [23], for which the hematocrit value was divided in 3 and expressed in g/dL.

### Determination of the effective dose of the quinoa flour of the Negra Collana variety and kanihua flour of the Ramis variety

10 groups (n = 3) of rats with experimental anemia were formed, they were administered orally by orogastric cannula quinoa flour and kanihua flour dissolved in vitamin C every 24 h. Quinoa flour was administered to 3 groups at doses of 180 mg/Kg, 360 mg/Kg and 460 mg/Kg, 3 groups received kanihua flour at doses of 180 mg/Kg, 360 mg/Kg, and 460 mg/Kg, in the evaluation of each flour there was 1 control group formed by anemic rats that received vitamin C and 1 control group formed by healthy rats with vitamin C. For 8 weeks, with a single blood draw per day.

### Evaluation of the antianemic activity of the quinoa flour of the Negra Collana variety and the kanihua flour of the Ramis variety

6 groups of rats (n = 6) were formed; they were administered orally by orogastric cannula, the flours dissolved in vitamin C every 24 h, Group A formed by anemic rats were administered 360 mg/Kg of quinoa flour. Group B formed by anemic rats was administered 360 mg/Kg of kanihua flour. Group C formed by healthy rats was administered 360 mg/Kg of quinoa flour. Group D formed by healthy rats was administered 360 mg/Kg of kanihua flour. Group E formed by anemic rats was administered vitamin C. Group F formed by healthy rats was administered vitamin C. The evaluation of the antianemic activity was carried out for 12 weeks, with a single blood draw per day.

### Ethical considerations

Due to the few previous studies and the absence of simulation models, the use of animals was essential to carry out this research. The National Institute of Health's recommendations for the care and use of laboratory animals in the aspects of food supply and cleaning were met [20]. The Ethics Committee approved the research of the Norbert Wiener Private University.

### Statistical analysis

We were performed with the statistical program STATA version 11.0 (Stata Corp LP, Collee Station, Texas). For the differential analysis of means, ANOVA and chi-square tests were used, the 95% confidence interval was used, and the value of *p* ≤ 0.05 was statistically significant. Analyzes were carried out in triplicate, expressed as the mean (µ) ± standard error of the sample (SEM).

## Results

### Proximal composition

The extruded flour of quinoa variety Negra Collana and the extruded flour of kanihua variety Ramis presented protein content of 22.0 ± 0.1% and 16.2 ± 0.1%, respectively. There was a higher protein content in quinoa flour than kanihua in 5.8%. In addition, the moisture and ash content was higher in the kanihua flour, but the carbohydrate content was lower than the quinoa flour (Table 1). About the content of micronutrients such as iron mineral, a higher content was observed in quinoa flour, followed by kanihua, kiwicha, corn, wheat, and rice.

**Table 1:** Proximal composition of the quinoa flour of the Negra Collana variety and kanihua flour of the Ramis variety compared to other grains

Parameters	Quinoa Negra Collana variety	Kanihua Ramis variety	Kiwicha*^a^	Rice*^b^	Wheat*^c^	Yellow corn*^d^
Proteins (%)	22.0 ± 0.1	16.2 ± 0.1	14.5	7.6	14.3	10.5
Moisture (%)	10.5 ± 0.0	11.6 ± 0.1	11.5	12.27	10.26	7.45
Ash (%)	2.9 ± 0.0	7.4 ± 0.0	2.3	0.27	1.6	1.6
Lipids (%)	3.5 ± 0.1	10.0 ± 0.0	5.7	2.2	2.1	5.36
Fiber (%)	7.1 ± 0.1	3.8 ± 0.0	4.4	0.9	2.5	2.7
Carbohydrates (%)	51.1 ± 0.0	50.9 ± 0.1	63.3	81.1	72.57	70.91
Iron (mg)/100 g	19.8^*a,b^	17.6^*a,b^	9.5	2.8	4.6	4.8
Calcium (mg)/100 g	94.0^*a,b^	171.0^*a,b^	176.0	23.0	48.0	48.3
Magnesium (mg)/100 g	204.0^*a,b^	165.0	244.0	157.0	152.0	107.9
Zinc (mg)/100 g	7.41^*a,b^	10.4	3.7	1.8	3.3	4.6
Phosphorus (mg)/ 100 g	403.6^*a,b^	387.0^*a,b^	453.0	325.0	387.0	299.6

Data expressed on a dry weight basis

*References: ^a^Mujica and Chura [7], ^a^Repo-Carrasco [15], ^b,c^FAO [8], ^b,c,d^Arisaca [25], ^b,c,d^Pereira[26]

### Determination of the acute toxicity of the quinoa flour of the Negra Collana variety and the kanihua flour of the Ramis variety

The administration of quinoa flour and kanihua flour up to the limit dose of 15000 mg/Kg for seven days did not cause the death of any of the experimental animals. Decreased motor activity, Straub tail sign, piloerection, skin itching, and hyperventilation were observed in rats receiving quinoa flour at a dose of 9000–15000 mg/Kg. However, the rats that received kanihua flour showed the same signs and symptoms but with the dose of 7000–15000 mg/Kg. This was observed within 30–60 min of dosing, and then rats showed full recovery and normal behavior within seven days of observation. Thus, in the administration of quinoa flour and kanihua flour, it was observed that the mean Lethal Dose (LD_50_) would be more than 15000 mg/Kg.

### Pathological analysis

**Fig. 1:**
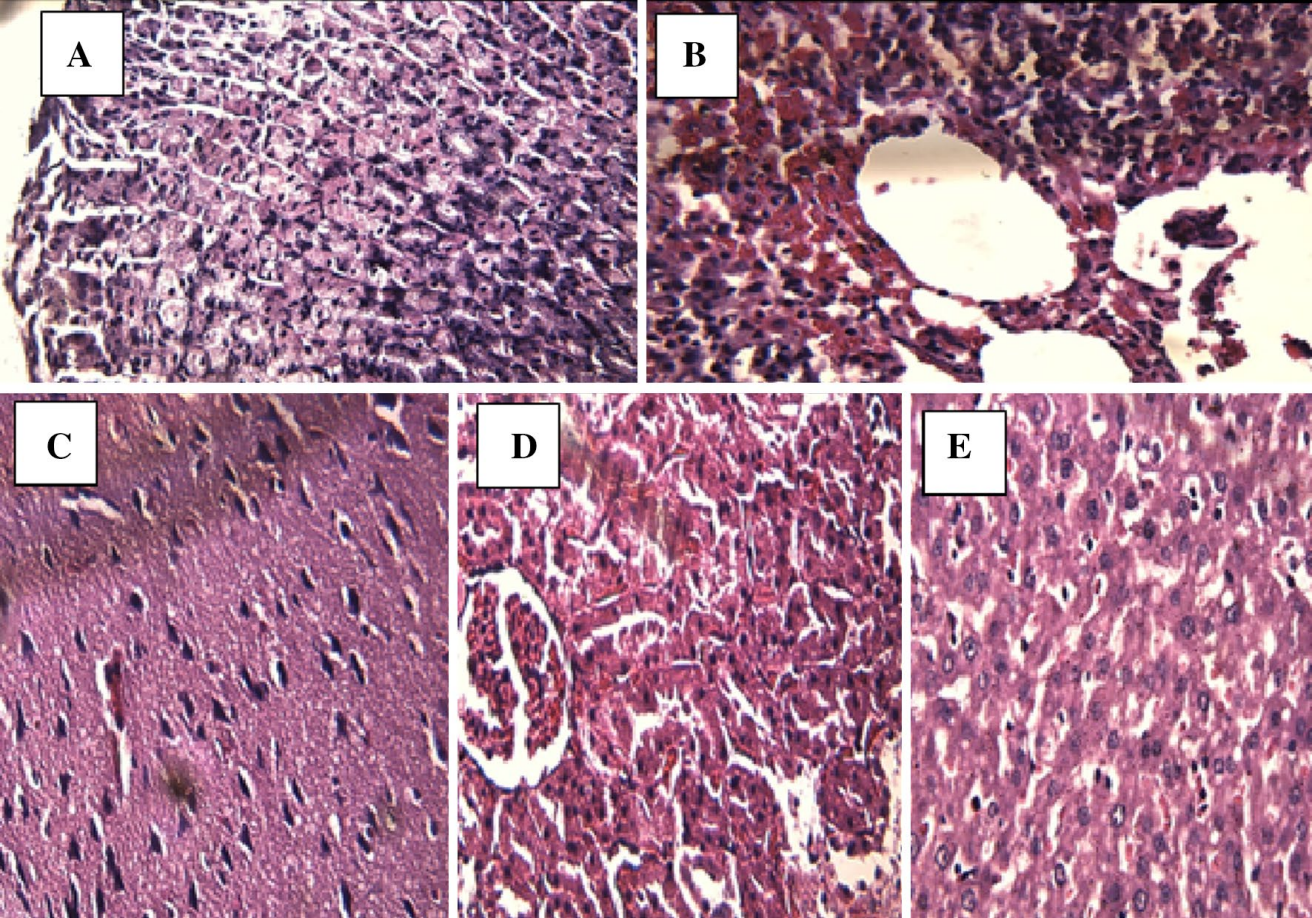
Histopathological analysis of rat organs from the acute toxicity test when quinoa flour, the Negra Collana variety, is administered orally. **A** Stomach. **B** Lung. **C** Brain. **D** Kidney. **E** Liver

During the necropsies, the macroscopic description of the extracted organs was made, such as the stomach, brain, liver, lung, and kidney of all the experimental animals of the acute toxicity test. The weight did not present significant differences with the control group, and they did not present any alteration related to the treatment of quinoa flour and kanihua flour. Subsequently, during the evaluation of the histopathological sections of the organs, no morphophysiological alterations were observed, maintaining the diameter and characteristics of each organ as usual; there was no inflammation or internal hemorrhage in any of the doses evaluated in the different organs with the administration of quinoa and kanihua flour (Fig. 1). This analysis confirmed the absence of toxicity of quinoa flour and kanihua flour observed with the acute toxicity test described above.

### Induction of experimental anemia

**Fig. 2:**
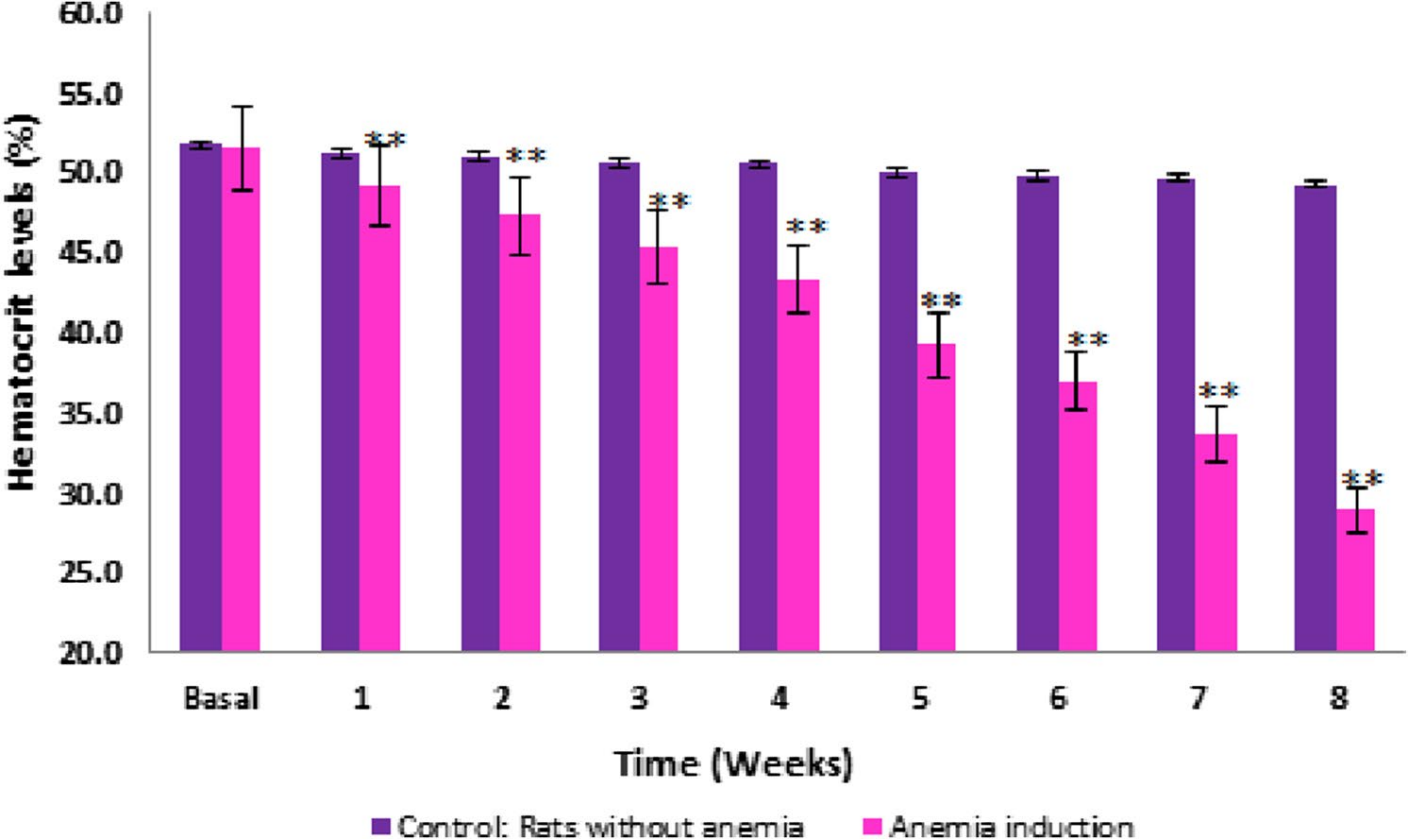
Hematocrit levels (%) of rats induced to experimental anemia for eight weeks. A significant decrease is observed from the second week of induction, obtaining less than 33% of hematocrit in the eighth week. Each point represents the mean ± SEM of three rats, *p ≤ 0.05, **p ≤ 0.01

The average hematocrit levels of healthy rats or the control group were between 51.7 ± 0.4% of baseline and 49.3 ± 0.2% in the eighth week. The rats subjected to experimental induction of anemia showed a baseline of 51.6 ± 0.6%, in the first week of induction 49.2 ± 0.5% was recorded, the fourth week of 43.3 ± 0.2%, and finally in the eighth week of 28.9 ± 0.3% showing a significant gradual decrease in hematocrit levels. Thus, a decrease of 22.7 ± 0.0% from baseline was observed compared to the eighth week (*p* ≤ 0.05) (Fig. 2). In addition, the anemic animals presented the classic signs and symptoms of anemia such as lethargy, hair loss, increased sleep, weight, and length loss. Anemic rats were considered those with hematocrit levels less than 33% of hematocrit and less than 11 g/dL of hemoglobin.

### Determination of the effective dose of the quinoa flour of the Negra Collana variety and kanihua flour of the Ramis variety

**Fig. 3:**
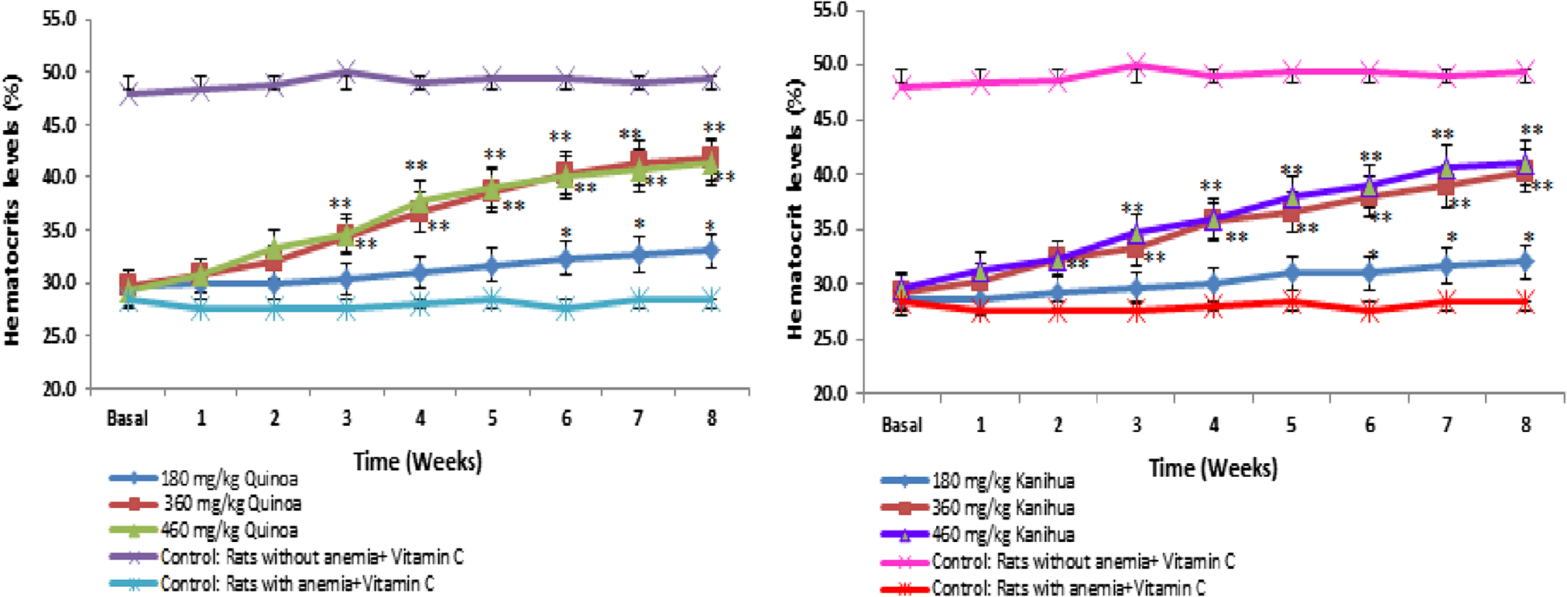
Hematocrit levels (%) of anemic rats when orally administered quinoa flour of the Negra Collana variety (left), and kanihua flour of the Ramis variety (right) at different doses for eight weeks. The 360 mg/kg dose of quinoa flour and kanihua flour significantly increased hematocrit levels to levels close to control rats without anemia. Each point represents the mean ± SEM of three rats, *p ≤ 0.05, **p ≤ 0.01

Eight weeks, the dose of 180 mg/Kg, 360 mg/Kg, and 460 mg/Kg of quinoa flour and kanihua flour were evaluated. In the evaluation of quinoa flour with the dose of 360 mg/Kg, the basal presented the average of the hematocrit levels of 29.7 ± 0.3%, the second week of 32.0 ± 0.6%, week five of 38.7 ± 0.3%, and in the eighth week of 41.7 ± 0.6% (*p* ≤ 0.05), with a significant increase of 12 ± 0.0% from baseline compared to the eighth week. With the 460 mg/Kg dose, the mean basal hematocrit levels were 29.3 ± 0.3%, the second week was 33.3 ± 0.7%, the fifth week was 39.0 ± 0.6%, and the eighth week was 41.3 ± 0.3% (*p* ≤ 0.05). There were no significant differences between the hematocrit levels obtained with the dose of 360 mg/Kg and 460 mg/Kg. In addition, an increase in hematocrit levels of 13.4 ± 0.0% and 13.0 ± 0.0% was observed compared to the control group of anemic rats, respectively (Fig. 3, left). Similar behavior was observed with the kanihua flour, with the dose of 360 mg/Kg it showed the baseline of 29.3 ± 1.0%, week two of 32.3 ± 0.3%, week five of 36.7 ± 0.3%, and week eight of 40.3 ± 0.3% (*p* ≤ 0.05), with a significant increase of 11 ± 0.0% from baseline compared to the eighth week. With the dose of 460 mg/Kg, the mean basal hematocrit levels were 29.7 ± 0.3%, the second week was 32.3 ± 0.3%, the fifth week was 38.0 ± 0.6%, and the eighth week was 41.0 ± 0.6% (*p* ≤ 0.05). There were no significant differences between the hematocrit levels obtained with the dose of 360 mg/Kg and 460 mg/Kg. In addition, hematocrit levels were 12.0 ± 0.0% and 12.7 ± 0.0% compared to the control group of anemic rats, respectively (Fig. 3, Right). Both flours with the dose of 180 mg/Kg in the eighth week showed an average of 32.0 ± 0.0%. Therefore, the dose of 360 mg/Kg of quinoa flour and kanihua flour was considered the optimal dose to be used in subsequent evaluations.

### Evaluation of the antianemic activity of the quinoa flour of the Negra Collana variety and the kanihua flour of the Ramis variety

**Fig. 4:**
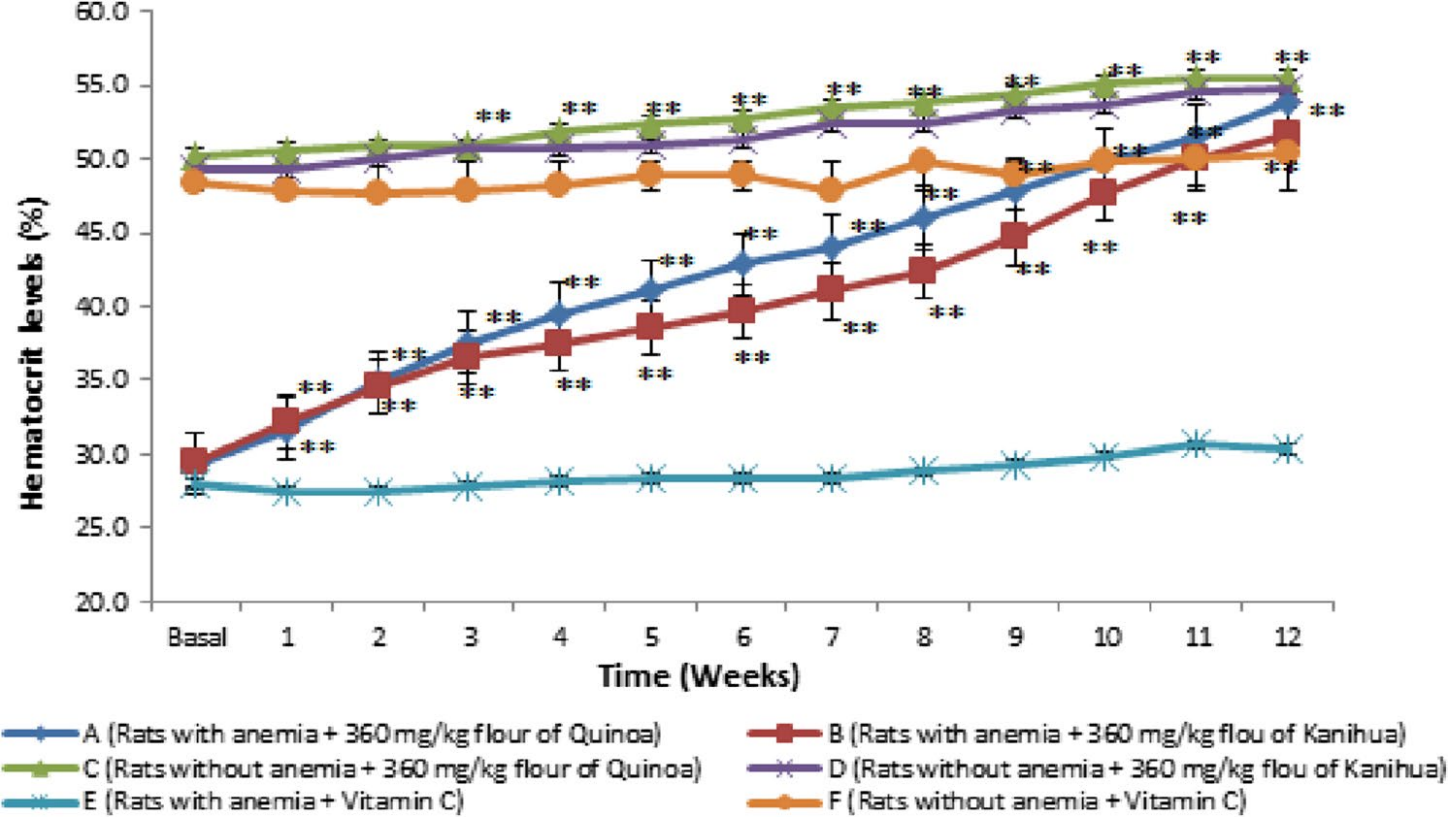
Hematocrit levels (%) during twelve weeks of treatment of the quinoa flour of the Negra Collana variety and kanihua flour of the Ramis variety orally every 24 h. Rats with anemia that were administered 360 mg/kg Quinoa flour Negra Collana variety (Group A), Rats with anemia that were administered 360 mg/kg Kanihua flour Ramis variety (Group B), Rats without anemia that were administered 360 mg/kg Quinoa flour Negra Collana variety (Group C), Rats without anemia that were administered 360 mg/kg Kanihua flour Ramis variety (Group D), Rats with anemia that were administered Vitamin C (Group E), Rats without anemia that were administered Vitamin C (Group F). Each point represents the mean ± SEM of three rats, *p ≤ 0.05, **p ≤ 0.01

The variations of the hematocrit levels obtained per week were observed during twelve weeks of treatment with the dose of 360 mg/Kg every 24 h orally in the six groups evaluated (Fig. 4). The anemic rats treated with the quinoa flour and vitamin C (Group A) started with a low basal mean hematocrit level of 29.3 ± 0.2%, in the second week, it was 34.8 ± 0.2% (*p* ≤ 0.05), in the fifth week they showed 41.0 ± 0.7% (*p* ≤ 0.05), in the eighth week 46.0 ± 0.6% (*p* ≤ 0.05), in the tenth week 49.8 ± 0.3% (*p* ≤ 0.05) and finally in the twelfth week 53.8 ± 0.3%; that is, an increase of 24.5% compared to baseline and 23.5% compared to control (Group E) was shown (*p* ≤ 0.05). The anemic rats treated with kanihua flour and vitamin C (Group B) showed a baseline of 29.5 ± 0.3%, in the second-week hematocrit levels of 34.5 ± 0.5% (*p* ≤ 0.05) were observed, in the fifth week showed 38.5 ± 0.9% (*p* ≤ 0.05), in the eighth week 42.3 ± 0.4% (*p* ≤ 0.05), in the tenth week 47.7 ± 0.8% (*p* ≤ 0.05) and finally in the twelfth week 51.7 ± 0.3%; thus, an increase of 22.2% compared to baseline and 21.4% compared to control (Group E) was shown (*p* ≤ 0.05). In this way, the antianemic activity of quinoa and kanihua flours, respectively, is shown in Group A and Group B; there was a more significant increase in hematocrit levels with quinoa flour than with kanihua from week five of treatment (*p* ≤ 0.05).

Meanwhile, the rats without anemia or healthy that received quinoa flour and vitamin C (Group C) presented hematocrit levels between 50.2 ± 0.2% at baseline and 55.5 ± 0.2% in the twelfth week, an increase of 5.3 ± 0.0% was shown compared to baseline and 25.2 ± 0.0% compared to control (Group E) (*p* ≤ 0.05). In rats without anemia or healthy that received kanihua flour and vitamin C (Group D) presented hematocrit levels between 49.3 ± 0.3% at baseline and 54.8 ± 0.1% in the twelfth week, there was an increase of 5.5 ± 0.0% in compared to baseline and 24.5 ± 0.0% compared to control (Group E). Thus, an increase in hematocrit levels and improved nutritional status were observed in Group C and Group D. In the anemic rats treated with vitamin C (Group E), hematocrit levels were observed between 28.0 ± 0.6% of baseline and 30.3 ± 0.8% in the twelfth week, showing the experimental anemia induced during the twelve weeks of the study. In the same way, in the rats without anemia or healthy that received only vitamin C (Group F), they presented normal blood glucose levels throughout the study between 48.3 ± 0.7% of the baseline and 50.3 ± 0.3% in the twelfth week; thus, it was evidenced that the food and water throughout the investigation did not influence the evaluation of the hematocrit levels.

It was observed that the quinoa flour increased significantly by 25.2% compared to the control group formed by anemic rats without treatment (Group E) and exceeded the hematocrit level of the control group of healthy rats without treatment by 5.2% treatment (Group F). Kanihua flour increased significantly by 24.5% compared to the control group formed by anemic rats without treatment (Group E) and exceeded the hematocrit level of the control group of healthy rats without treatment by 4.5% (Group F). Therefore, the oral administration of 360 mg/kg every 24 h of extruded quinoa flour and extruded kanihua flour concomitantly with vitamin C during the twelve weeks of treatment showed antianemic activity in anemic rats by significantly increasing hematocrit levels and increased hematocrit levels in rats without anemia.

Table 2 shows the data for hemoglobin, weight, and length of rats from the baseline and twelfth week when they are orally administered 360 mg/Kg of quinoa flour and kanihua flour. In anemic rats from Group A and Group B, there was an increase of 8.2 g/dL and 7.4 g/dL of hemoglobin, 65.8 g and 59.2 g of weight; 6.8 cm and 5.7 cm in length, respectively (*p* ≤ 0.05). In Group C and Group D rats without anemia, an increase of 1.8 g/dL of hemoglobin, 37.7 g, and 21.7 g of weight was shown in both groups; 4 cm and 3.9 cm in length, respectively. Nevertheless, in anemic rats that only received vitamin C (Group E), they maintained constant hemoglobin levels, weight, and length, showing experimental anemia with characteristics of anemia such as low weight, length, hair loss, lethargy, and increased sleep. Rats without anemia or healthy that received vitamin C (Group F) showed similar hemoglobin, weight, and length levels at twelve weeks.

**Table 2:** Average hemoglobin levels (g/dL), weight (g), and length (cm) of *Holtzman* strain rats that received orally 360 mg/Kg of quinoa flour of the Negra Collana variety and kanihua flour of the Ramis variety for twelve weeks

Groups	Hemoglobin levels (g/dL)		Weight (g)		Length (cm)	
	Baseline	12 weeks	Baseline	12 weeks	Baseline	12 weeks
A	9.7 ± 0.4	17.9 ± 0.3*	145.0 ± 0.6	210.8 ± 0.2*	34.3 ± 0.2	41.1 ± 0.7*
B	9.8 ± 0.2	17.2 ± 0.2*	138.3 ± 0.7	197.5 ± 0.3*	35.1 ± 0.7	40.8 ± 0.9*
C	16.7 ± 0.4	18.5 ± 0.2*	191.8 ± 0.1	229.5 ± 0.7*	38.0 ± 0.0	42.0 ± 0.8*
D	16.4 ± 0.2	18.2 ± 0.3*	190.3 ± 0.4	212.0 ± 0.4*	37.1 ± 0.7	41.0 ± 0.8*
E	9.3 ± 0.4	10.1 ± 0.6	144.6 ± 0.3	149.8 ± 0.3	34.3 ± 0.0	35.6 ± 0.0
F	16.1 ± 0.5	16.7 ± 0.2	190.6 ± 0.2	199.0 ± 0 .8	37.6 ± 0.5	39.5 ± 0.5

A, anemic rats treated with 360 mg/kg quinoa flour; B, anemic rats treated with 360 mg/kg kanihua flour; C, healthy rats treated with 360 mg/kg quinoa flour; D, healthy rats treated with 360 mg/kg kanihua flour; E, Anemic rats + vitamin C; F, Healthy rats + vitamin C. Each point represents the mean ± SEM of three rats, *p ≤ 0.05

## Discussion

Anemia has a current and future impact on the economic productivity of our country and the world because it causes low cognitive and labor development in patients. In terms of productivity, anemia affects a range of 5% to 17% in manual jobs; and 4% in all other types of work in youth and adults [2, 4]. Thus, quinoa flour (*Chenopodium quinoa* Willd) of the Negra Collana variety and kanihua flour (*Chenopodium pallidicaule* Aellen) of the Ramis variety are antianemic nutraceutical alternatives due to their high protein content, the balance of essential amino acids, good content of vitamins and minerals such as iron; that can be used in different economic levels of the population, due to the accessibility of cost compared to animal proteins, production, and ease of preparation. Obtaining flour from Andean grains of quinoa and kanihua allowed obtaining particles of 0.250 mm that facilitated oral administration. The grain extrusion process is used to improve physical and chemical characteristics, to have better mineral bioavailability such as iron, destruction of anti-nutritional factors such as saponins, increased digestibility of macronutrients, and is one of the best conservation methods [25]. Different techniques have been used to remove them, such as the wet method carried out by the Andean inhabitants who constantly wash the seeds with water and rub them with their hands. At an industrial level, the dry method is used; they use extrusion machines to eliminate the saponins partially, and the flour obtained has optimal functional, nutritional, and sensory characteristics [7, 15]. Mujica et al*.* [14] reported pearling quinoa as the most efficient method of removing saponins, and it consists of scarifying, washing, and drying under innocuous conditions. In our study, the extrusion procedure was mainly used to partial removal saponins that provide the characteristic bitter taste of quinoa seeds; and the kanihua saponins that do not have a bitter taste. The nutritional value of food in composition and content is determined with proximal analysis, which allows obtaining an optimal final product and quality control [15]. The Andean grains of quinoa and kanihua are considered complete due to their high biological quality and nutritional value, with a high amount of protein and optimal balance of essential amino acids compared to other grains [7, 8]. Thus, the amount of protein of quinoa variety Negra Collana was 22.0 ± 0.1% and of kanihua variety Ramis was 16.2 ± 0.1%; higher values compared to other varieties such as Quillahuaman INIA quinoa with 13%, INIA 415 Pasankalla with 16.8%, Altiplano 14% and kanihua ILLPA INIA with 15.7% [7, 15], and other grains such as wheat with 14.3%, rice 7.6%, corn 10.5%, beans 28.0% [5]. The fat content in the 10% kanihua is higher than the 3.5% quinoa and other grains; the fiber content in both quinoa and kanihua is more significant than 7.1%, 3.8%, respectively, compared to kiwicha of 4.4% and wheat of 2.5% [7, 8]. The moisture, ash, fat, and carbohydrate content of the quinoa and kanihua flour in our research were within the range reported in some cereals according to Mujica and Chura [7], Mujica et al. [14], and Repo-Carrasco [15]. In the extruded flour of quinoa variety Negra Collana INIA 420, they reported a high iron content of 19.8 mg, as well as the extruded flour of kanihua variety Ramis of 17.6 mg, unlike other grains such as kiwicha with 9.5 mg, rice 2.8 mg, and wheat 4.6 mg [8, 25, 26]. It is essential to mention that the extrusion technique allows the best biodisposition of minerals such as iron.

Continuing with the investigation, in the results of acute toxicity, signs and symptoms of decreased motor activity, Straub tail, piloerection, skin itching, and hyperventilation were observed with the dose of 7000–15000 mg/Kg in the administration of kanihua flour, and 9000–15000 mg/Kg with quinoa flour. This were observed within 30–60 min of dosing, and then rats showed full recovery and normal behavior within seven days of observation, which is due to a large amount of flour administered orally as a single dose. So, the administration of quinoa flour and kanihua flour were harmless with no toxicity signs and symptoms; it was observed that the mean Lethal Dose (LD_50_) would be more than 15000 mg/Kg. In the investigation by Tillán et al. [27], acute oral toxicity was carried out with a single maximum dose of 2000 mg/Kg of the lyophilized aqueous extract of *Ocimum tenuiflorum* L. in rats, showing no toxic signs after administration in the fourteen days of evaluation. However, Orellana-Cuellar et al*.* [28] evaluated the acute toxicity of *Aleurites moluccana* orally in rats and with the maximum single dose of 2000 mg/Kg described moderate and intense apathy, decreased movement, decreased reflexes, piloerection, hair loss and decreased eye-opening as signs of toxicity in the fourteen days of evaluation. Moscoso-Mujica et al. [16] evaluated the acute toxicity according to the Williams criteria of the aqueous extract of *Argyrochosma nivea* (Poir.) Windham in mice, reporting decreased motor activity, Straub tail, and hyperventilation from a dose of 9000 mg/Kg.

The anatomopathological examination corresponding to the external and macroscopic inspection of the organs of the acute toxicity rats did not show alterations related to the treatment of quinoa flour and kanihua flour. Then, in the microscopic evaluation of the histopathological sections of the different organs such as the stomach, lung, brain, kidney, and liver, no morpho-physiological alterations were observed in the evaluated organs compared to the control group, maintaining the standard diameter and characteristics of each organ, without the presence of inflammation and internal bleeding. In the investigation by Rodríguez et al. [29] that evaluated the toxicity of single administration of the product QT2B21 in Sprague Dawley rats, they did not report alterations in organs or cavities in the pathological examination; in the histopathological examination, they reported differences significant differences in the weight of male rats with the control. Paixao et al*.* [30] reported that the organs histopathological analysis after the toxicity test shows hemorrhage alterations or injury due to extracts or medications; it is a widely used technique to corroborate the findings of the tests of acute toxicity. In the investigation, the anatomopathological analysis showed the absence of toxicity of both flours, corroborating the acute toxicity test; thus, the consumption of extruded flour of quinoa seeds variety Negra Collana and extruded flour of kanihua seeds variety Ramis are safe and innocuous foods up to high doses. In other investigations such as Ramírez et al*.* [31] reported that the consumption of quinoa grain is safe; Rojas et al*.* [5] describe the safe and innocuous consumption of quinoa in different forms of consumption since pre-Inca times. Likewise, Repo-Carrasco [15] and Moscoso-Mujica et al*.* [18] report the safe and innocuous consumption of kanihua in Andean inhabitants. The FAO [8] indicates that the Andean grains quinoa and kanihua as functional foods have a high content of essential amino acids and are safe and highly nutritious foods.

Anemia is a disorder of the reduced amount of hemoglobin that is located in the red blood cells and of the decrease in the transport of oxygen from the lungs to the tissues and of carbon dioxide in the opposite direction; the number of red blood cells is of no value in defining anemia because there may be red blood cells that are devoid of hemoglobin. The globular protein of hemoglobin is a 64 kDa tetramer formed by four polypeptide chains, two of the α-globin type of 141 amino acids and two of the β-globin type of 146 amino acids; each globin contains a heme prosthetic group formed by an iron atom that it transports oxygen and a porphyrin ring [1, 2, 32]. Iron biomarkers are essential tools to predict the health and proper functioning of the metabolism due to the importance of this metal in various disorders such as anemia. In clinical practice, biomarkers are used routinely to determine iron nutrition status, including tests of hematocrit, hemoglobin, and indirectly weight and length [4, 22, 33].

Subsequently, in the results of the induction of experimental anemia, the average hematocrit level of 28.9 ± 0.3% was shown in the eighth week compared to the control group of 51.7 ± 0.4%, accompanied by signs and symptoms such as hair loss, thinness, pale tail, legs and ears, lethargy in their movements and excessive sleepiness that are characteristic of experimental anemia. Animals with experimental anemia were considered with hematocrit levels less than 33% and hemoglobin less than 11 g/dL [22, 34]. The loss of blood extracted by the saphenous vein recurrently for a prolonged period causes the loss of red blood cells and loss of hemoglobin in an amount more significant than its production and therefore causes experimental anemia [1, 33]. Sánchez [35] reported similar signs and symptoms in animals with experimental anemia such as Anaya et al*.* [36]; both investigations reported experimental anemia in rats with hematocrit levels below 33%. When there is a loss, or the body needs to produce more red blood cells, the hormone erythropoietin produced in the kidneys sends the signal to the bone marrow and the heme group that is synthesized in all tissues, mainly in the bone marrow and liver, are incorporated into hemoglobin and cytochromes, respectively [37]. The heme group, formed by ferrous iron and a tetrapyrrolic ring, is the main factor regulating the rate of globin synthesis, which participates in the initiation of translation by blocking the action of the inhibitor of globin production, and plays a role important in the protein synthesis of mammalian erythrocytes, liver, and brain tissue; any anomaly in these levels will produce diseases such as anemia [32, 37].

In the evaluation of the effective dose of antianemic activity, it was observed with the administration of 360 mg/Kg of quinoa flour the baseline of 29.7 ± 0.3% and the hematocrit levels increased from the second week, in the eighth week, a significant increase of 12 ± 0.0% was shown, reaching hematocrit levels of 41.7 ± 0.6% (*p* ≤ 0.05). Similarly, the dose of 460 mg/Kg presented hematocrit levels of 41.3 ± 0.3% in the eighth week (*p* ≤ 0.05). With the kanihua flour, the basal dose of 360 mg/Kg was 29.3 ± 1.0%, increasing hematocrit levels from the second week and the eighth week up to 41.0 ± 0.3% (*p* ≤ 0.05). No significant differences were shown in the increase in hematocrit levels with the dose of 360 mg/Kg and 460 mg/Kg in both flours during eight weeks of treatment. The dose of 360 mg/Kg was considered of quinoa flour and kanihua flour as the optimal dose to be used in subsequent evaluations for presenting better performance in effectiveness and cost. Atata et al*.* [38] reported a higher antianemic effect in anemic rats with a dose of 500 mg/Kg of *Cnidoscolus aconitifolius*, and the investigation by Tillan et al. [27] showed a better antianemic effect with a diet composed of 15 mg/Kg of iron associated with 750 mg/Kg of *Cassia grandis* L.

In evaluating the antianemic activity of the quinoa flour of the Negra Collana variety and the kanihua flour of the Ramis variety, a dose of 360 mg/Kg was used orally every 24 h for twelve weeks of treatment. Anemic rats treated with quinoa flour and vitamin C (Group A) showed a significant increase in hematocrit levels of 24.5 ± 0.5% compared to baseline and 23.5 ± 0.0% compared to control (Group E). Anemic rats treated with kanihua flour and vitamin C (Group B) showed a significant increase in hematocrit levels of 22.2 ± 0.3% compared to baseline and 21.4 ± 0.0% compared to control (Group E) (*p* ≤ 0.05), the average hematocrit levels obtained in twelve weeks of treatment with quinoa flour was 53.8 ± 0.1% and with kanihua flour 51.7 ± 0.1%, evidencing a significant increase in hematocrit levels. Also, it was observed in rats without anemia that received quinoa flour and vitamin C (Group C) an increase of 5.3 ± 0.0% compared to baseline and 25.2 ± 0.0% compared to control (Group E) (*p* ≤ 0.05). Rats without anemia that received kanihua flour and vitamin C (Group D) increased 5.5 ± 0.0% compared to baseline and 24.5 ± 0.0% compared to control (Group E) (*p* ≤ 0.05), in these two groups, showed that both meals in healthy animals increase hematocrit levels with optimal nutrition. Unlike the rats without anemia that only received vitamin C (Group F) maintained their hematocrit levels between 48.3 ± 0.7% and 50.3 ± 0.0%, in this group, it was shown that the food and water used throughout the investigation did not influence the results; and in the anemic rats treated only with vitamin C (Group E) their hematocrit levels were between 28.0 ± 0.6% and 30.3 ± 0.8%, showing experimental anemia throughout the investigation. Likewise, the increase in weight and length was appreciated in the animals that received the quinoa and kanihua flour, showing the nutritional value of these two Andean grains as functional foods and nutraceuticals (*p* ≤ 0.05).

For all the results obtained in the investigation, it was indicated that the oral administration of 360 mg/Kg every 24 h of the quinoa flour of the Negra Collana variety and the kanihua flour of the Ramis variety concomitantly with vitamin C presented antianemic activity by increasing significantly increased hematocrit levels in anemic rats compared to control groups during twelve weeks of treatment. Also, improvement in hematocrit levels and nutritional status was observed in healthy rats; in addition, a better antianemic effect could be seen with quinoa flour compared to kanihua flour. There are few investigations of the Andean quinoa grain such as that reported by Anaya et al*.* [36] evaluated biscuits with white quinoa flour: 36.2 g: 36.2 g (w/w) bovine blood in anemic rats and showed an increase in hematocrit levels up to 47.1% (15.7 g/dL hemoglobin) for five weeks, indicated that the content of macro and micronutrients of quinoa enriched with heme iron improved the anemic state and symptomatology of anemia. Amaro-Terrazos et al*.* [34] evaluated quinoa consumption in mice with iron deficiency anemia, reporting that 20 g/day of quinoa extract increased hematocrit by 6.3% (2.1 g/dL hemoglobin) compared to the control group for seven weeks; concluded by indicating that under experimental conditions, quinoa has an antianemic effect, supported by the results of hemoglobin levels. Unlike few studies of kanihua in anemia, such as the one by Novak et al. [39] that evaluated the administration of kanihua: vitamin C (50:100 w/w) in non-pregnant and non-lactating women at risk of anemia in Puno, Peru; observed that after seven weeks of oral treatment, hemoglobin and hematocrit levels increased significantly compared to the control group; they indicated that the high amount of iron in kanihua improved the anemic state with notable improvement in symptoms. In other investigations of antianemic activity, Robles et al. [40] evaluated fermented goat's milk in anemic *Wistar* rats for 30 days, and their results showed hepatosomatic index with an increased body weight of 62.7 g, a liver weight of 2.5 g, liver weight/body weight of 0.44% and liver iron content of 55.2 µg/g of dry weight; compared to the control group (*p* ≤ 0.05). They observed that iron deficiency decreased weight gain, lean mass, and body fat, indicating lower energy stores. Therefore, fermented goat milk with 20% protein and 3.5% minerals more efficiently recovered the iron level during iron deficiency recovery, decreased adiposity, and increased energy expenditure. Recovery from anemia and nutritional status were observed. Darwish et al*.* [41] evaluated functional foods such as yogurt from milk fermentation, fortified with iron in bovine serum albumin nanoparticles, as an alternative to improve iron deficiency anemia. In addition, the investigation indicated the benefits of the nutritional compounds of the milk yogurt for the protein and iron content; the whey albumin nanoparticle also increased the protein content. They showed that the functional food restored hemoglobin to 16.53 g/dL, iron 109, 25 µg/dL, ferritin 33.25 µg/dL, and total protein 8.6 g/dL during four weeks of feeding in anemic albino rats.

The antianemic activity shown in the present investigation of extruded quinoa flour and extruded kanihua flour could be due to the high amount of iron of 19.8 mg and 17.6 mg, such as the high protein content of 22% and 16.2%, respectively. Which makes it possible to have endogenous nitrogen sources for subsequent uses in the synthesis of proteins such as hemoglobin, ferritin, and other proteins; as well as the balanced composition of essential and non-essential amino acids, vitamins, fatty acids, and fiber that improve the nutritional status of the anemic patient. As reported, iron is an essential mineral used in several enzymatic functions that participate in DNA synthesis, oxygen transport, energy metabolism, and other functions [33, 42]. The body has an average iron content of 3–4 g, distributed in erythrocytes, liver, bone marrow, muscles and other tissues, and 75% is used to form part of hemoglobin, myoglobin, and other enzymes, and 0.5 g of iron is deposited in the liver; but, excess iron is related to oxidative stress [38, 42]. Thus, an adequate regulatory system for this mineral is necessary; because the absorption of iron is 1 to 2 mg per day, which varies depending on the body's needs, such as the activity of the bone marrow, hemoglobin concentration, and other activities of the organism [33]. Plant-derived iron is called non-heme iron, and it is mainly in the oxidized form (Fe^3+^); it is absorbed in the duodenum and the divalent metal transporter 1 (DMT1) allows it to enter the enterocyte. For this, it must be reduced to its ferrous form (Fe^2+^) in the stomach through gastric acid and the duodenum, with the help of the cytochrome B reductase enzyme located in the intestinal brush border [32, 33]. In our study, the concomitant administration of vitamin C or ascorbic acid was proposed, which allowed improving the reduction of ferric iron (Fe^3+^) to allow better absorption of non-heme iron from quinoa and kanihua. Iron enters the cytoplasm and is distributed according to body needs; it is stored in the protein ferritin with 4500 iron atoms; then, transferrin transports iron from the intestinal lumen and from that obtained in the degradation of hemoglobin. When ferritin binds to its receptor on the cell membrane, it forms a complex and enters the endosome, and will release Fe^3+^at acidic pH from the ATP-dependent proton pump, to be reduced to Fe^2+^ and allow it to exit to the cytosol through DMT1 and become part of the labile iron pool. Thus, iron regulation will depend on the optimal amount of exogenous iron, such as that provided by extruded flour from quinoa seeds of the Negra Collana variety and kanihua flour of the Ramis variety. In addition, to provide a high content of proteins and amino acids, nitrogen sources to form different endogenous proteins, unsaturated fatty acids that participate in important and different metabolic routes, vitamins, and other minerals that participate as cofactors in metabolic pathways.

## Conclusions

The high protein content of quinoa variety Negra Collana flour and kanihua variety Ramis flour of 22% and 16%, respectively; essential amino acids such as isoleucine 3.4 mg and 6.4 mg, leucine 6.1 mg and 6.1 mg, and lysine 5.6 mg and 5.7 mg, respectively. Minerals such as non-heme iron of 19.8 mg/100 g and 17.6 mg/100 g, respectively. Like the other nutritional components in the optimal and balanced quantity shown in the proximal analysis of these seeds, they are positioned as functional foods with a high biological value, similar to different animal proteins. The absence of toxicity and safety of the administration of both flours up to high doses allow their wide use. The significant increase in hematocrit levels of anemic rats up to 53.8 ± 0.05% and 51.7 ± 0.05% showed the antianemic activity of quinoa flour and kanihua flour, respectively (*p* ≤ 0.05); with the oral dose of 360 mg/Kg every 24 h. In addition, there was an improvement in weight and length and signs and symptoms of anemia; in healthy rats, moderate increases in hematocrit levels, weight, and length were observed. Therefore, extruded flour from quinoa seeds (*Chenopodium quinoa* Willd) variety Negra Collana and extruded flour from seeds of kanihua (*Chenopodium pallidicaule* Aellen) variety Ramis could be a natural nutraceutical alternative for the treatment of anemia.

## References

[1] WHO (World Health Organization) (2011) Hemoglobin concentrations to diagnose anemia and assess its severity. Geneva. (WHO/NMH/NHD/MNM/11.1). https://www.who.int/vmnis/indicators/haemoglobin_es.pdf Accessed 01 Mar 2022

[2] Kurz K (1996). Adolescent nutritional status in developing countries. Proc Nutr Soc.

[3] Horton S, Alderman H, Rivera JA (2008) Copenhagen consensus 2008 challenge paper hunger and malnutrition. Frederiksberg Denmark. https://www.copenhagenconsensus.com/sites/default/files/CP_Malnutrition_and_Hunger_-_Horton.pdf Accessed 15 Dec 2021

[4] MINSA. (Ministry of Health of Peru) (2017) Technical document: national plan for the reduction and control of maternal-child anemia and chronic child malnutrition in Peru: 2017–2021. Lima: MINSA RM No 249. http://bvs.minsa.gob.pe/local/MINSA/4189.pdf. Accessed 23 Nov 2021

[5] Rojas J, Ren G, Mujica A (2022). Quinoa: the sacred grain of the Incas.

[6] Mujica A, Jacobsen S (2006) Quinoa (*Chenopodium quinoa* Willd.) and its wild relatives. In: Morales M, Øllgaard B, Kvist L, Borch senius L, Balslev H (eds) Economic botany of the central andes, 1st edn. University of San Andres-La Paz, Bolivia, pp 449–457.

[7] Mujica A, Chura E (2012) Andean seeds and cereals cultivation. Academic Vice President and University Research Office, National University of the Altiplano-Puno, Peru

[8] FAO (Food and Agriculture Organization of the United Nations) (2011) Quinoa: ancient crop to contribute to world food security. Rome-Geneva. URL: https://www.fao.org/3/aq287s/aq287s.pdf. Accessed 05 Nov 2021

[9] Moscoso-Mujica G, Zavaleta A, Mujica A, Santos M, Calixto R (2017). Fractionation and electrophoretic characterization of (*Chenopodium pallidicaule* Aellen) kanihua seed proteins. Rev Chil J Nutr.

[10] Ranilla L, Apostolidis E, Genovese M, Lajolo F, Shetty K (2009). Evaluation of indigenous grains from the peruvian Andean region for antidiabetes and antihypertension potential using in vitro methods. J Med Food.

[11] Graf B, Poulev A, Kuhn P, Grace M, Lila M, Raskin I (2014). Quinoa seeds leach phytoecdysteroids and other compounds with anti-diabetic properties. Food Chem.

[12] Campos D, Chirinos R, Ranilla L, Pedreschi R (2018). Bioactive potential of Andean fruits, seeds, and tubers. Adv Food Nutr Res.

[13] Lin M, Han P, Li Y, Wang W, Lai D, Zhou L (2019). Quinoa secondary metabolites and their biological activities or functions. Molecules (Basel, Switzerland).

[14] Mujica A, Jacobsen S, Ortiz R, Canahua A, Apaza V, Aguilar P, Dupeyrat R (2002) Research in kanihua (*Chenopodium pallidicaule* Aellen). National University of the Altiplano-Postgraduate School-Investigation Institute-Puno-Peru

[15] Repo-Carrasco R (2011) Andean indigenous food crops: nutritional value and bioactive compounds-Department of Biochemistry and Food Chemistry-University of Turku, Turku-Finland

[16] Moscoso-Mujica G, Mujica A, Vegas C, Villena M, Alvizuri H (2017). Preclinical and clinical evaluation on the hypoglycemic actuvity of Inca sayre (Argyrochosma nívea (Poir) Windham) in type 2 diabetes mellitus. J Fitotherapy.

[17] Hu Y, Zhang J, Zou L, Fu C, Li P, Zhao G (2017). Chemical characterization, antioxidant, immune-regulating and anticancer activities of a novel bioactive polysaccharide from *Chenopodium quinoa* seeds. Int J Biol Macromol.

[18] Moscoso-Mujica G, Zavaleta A, Mujica A, Arnao I, Moscoso-Neira C, Santos M, Sánchez J (2021). Antimicrobial peptides purified from hydrolysates of kanihua (*Chenopodium pallidicaule* Aellen) seed protein fractions. Food Chem.

[19] AOAC (Official Methods of analysis) (2019). Method 920.14 and 992.24 (21st edn). Association of Official Analytical Chemists. Washington, USA. URL https://www.aoac.org/official-methods-of-analysis-21st-edition-2019/

[20] NIH (National Institute of Health) (2011) Guide for the care and use of laboratory animals (8th edn). The National Academy Press No. 85–23, USA

[21] Montalvo C (2010) Histological technique. Publishing UNAMMEDWeb. https://bct.facmed.unam.mx/wp-content/uploads/2018/08/3_tecnica_histologica.pdf Accessed 13 Nov 2022

[22] Delwatta S, Gunatilake M, Baumans V, Seneviratne M, Dissanayaka M, Batagoda S, Udagedara A, Walpola P (2018). Reference values for selected hematological, biochemical and physiological parameters of Sprague-Dawley rats at the animal house, faculty of medicine, university of Colombo, Sri Lanka. Animal Model Exp Med.

[23] Rodak B (2014) Hematology clinical principles and applications, 4th edn). Medical panamericana- Buenos Aires, Argentina

[24] Márquez M, Chacón J (2016). Determination of VSG: comparison of methods and microhaematocrit Wintrobe. Rev Salud Publica Bogota.

[25] Arisaca A (2016) Antioxidant capacity of three agroindustrial processes of quinoa (*Chenopodium quinoa* Willd) Ayara ecotype and INIA 420 Negra Collana variety and availability of lithium. [Degree Thesis, National University of Alptiplano Puno - Peru]. http://repositorio.unap.edu.pe/handle/UNAP/6592 Accessed 15 Oct 2021

[26] Pereira E, Encina-Zelada C, Barros L, Gonzales-Barron U, Cadavez V, Ferreira C (2019). Chemical and nutritional characterization of *Chenopodium quinoa* Willd (quinoa) grains: a good alternative to nutritious food. Food Chem.

[27] Tillán J, Rodríguez J, Gómez J, Pardo Z, Agüero S (2004). Antianemic activity *Cassia grandis* L. Cuban J Pharm.

[28] Orellana-Cuellar L, Montañez M, Moron I, Orellana A, Casildo L, Aguilar E, Barrutia J, Granda B, Sánchez W, Villanueva A (2014). Acute oral toxicity of *Aleurites moluccana* in *Sprague-Dawley* rats. CIMEL.

[29] Rodríguez J, Quesada W, Pérez R, Ávila A, Guzmán A, León G (2004). Evaluation of the toxicity by single administration of the product QT2B21 in *Sprague Dawley* rats. Rev Cubana Plant Med.

[30] Paixao A, Mancebo B, Regalado A, Chong D, Sanchez L (2017). Oral acute toxicity of a *Tephrosia vogelii* Hook (kalembe) ethanolic extrac. Salud Anim.

[31] Ramírez D, Ramírez E, Sáenz L (2016). Nutritional properties of quinoa and its paradoxes of exclusion and social inclusion in Peru (2011–2014). Social Inv.

[32] Salazar A, Sandoval A, Armendariz J (2016). Molecular biology: fundamentals and applications in the health sciences.

[33] Sermini C, Acevedo M, Arredondo M (2017). Biomarkers of metabolism and iron nutrition. Rev Peru Med Exp Salud Publica.

[34] Amaro-Terrazos J, Iparraguirre M, Jiménez A (2019). Effect of quinoa extract consumption on iron deficiency-induced anemia in mice. Rev Salud Publica (Bogota).

[35] Sánchez A (2014) Effect of folic acid supplementation in milk (goat or cow) on oxidative damage to biomolecules in recovery from iron deficiency anemia [Doctoral thesis, University of Granada, Institute of nutrition and food technology, Spain]

[36] Anaya B, De La Cruz E, Cóndor R, Espitia E, Navarro R, Rivera J (2020). Assessment of the formulations of anti-anemic biscuits with different contents of Quinoa and different contents in heminic iron, by reduction of anemia in *Holtzman* rats. Rev Bol Quim.

[37] Burmester T, Weich B, Reinhardt S, Hankeln T (2000). A vertebrate globin expressed in the brain. Nature.

[38] Atata J, Ayoola T, Ajadi A, Adamu S, Olatunji A, Biobaku K (2020). Antianemic effect of ethanol leaf extract of *Cnidosculus aconitifolius* on cyclophosphamide-induced anemia in rats. J Complem Integr Med.

[39] Novak W, Mujica A, Vogl C, Jacobsen S (2002) The effect of Canahua (*Chenopodium Pallidicaule* Aellen) on hemoglobin levels and iron status of rural women in risk of anemia in Puno, Peru. Dissertation, European Tropical Forest Research Network, Vienna-Austria

[40] Robles M, López I, Díaz J, Moreno J, Muñoz M (2020). Iron status, weight changes and body composition during recovery from anemia in an experimental model: effect of fermented goat or cow milk. Nutr Hosp.

[41] Darwish AMG, Soliman TN, Elhendy HA, El-Kholy WM (2001). Nano-encapsulated iron and folic acid-fortified functional yogurt enhance anemia in albino rats. Front Nutr.

[42] Aisen P, Enns C, Wessling-Resnick M (2001). Chemistry and biology of eukaryotic iron metabolism. Int J Biochem Cell Biol.

